# Rare Variants in Genes Encoding *MuRF1* and *MuRF2* Are Modifiers of Hypertrophic Cardiomyopathy

**DOI:** 10.3390/ijms15069302

**Published:** 2014-05-26

**Authors:** Ming Su, Jizheng Wang, Lianming Kang, Yilu Wang, Yubao Zou, Xinxing Feng, Dong Wang, Ferhaan Ahmad, Xianliang Zhou, Rutai Hui, Lei Song

**Affiliations:** 1 State Key Laboratory of Cardiovascular Diseases, Fuwai Hospital, National Center for Cardiovascular Disease, Chinese Academy of Medical Sciences and Peking Union Medical College, Beijing 100037, China; E-Mails: suming28@163.com (M.S.); jzwang@hotmail.com (J.W.); lianmingkang@126.com (L.K.); zouyb1973@gmail.com (Y.Z.); xinxing_feng@hotmail.com (X.F.); wangdongfw@gmail.com (D.W.); zhouxianliang0326@hotmail.com (X.Z.); 2 Intensive Care Unit, China Meitan General Hospital, Beijing 100028, China; E-Mail: wangyilu0908@163.com; 3 Division of Cardiovascular Medicine, Department of Internal Medicine, University of Iowa Carver College of Medicine, Iowa City, IA, USA; E-Mail: ferhaan-ahmad@uiowa.edu

**Keywords:** hypertrophic cardiomyopathy, muscle ring finger protein, rare variant, modifier

## Abstract

Modifier genes contribute to the diverse clinical manifestations of hypertrophic cardiomyopathy (HCM), but are still largely unknown. Muscle ring finger (MuRF) proteins are a class of muscle-specific ubiquitin E3-ligases that appear to modulate cardiac mass and function by regulating the ubiquitin-proteasome system. In this study we screened all the three members of the MuRF family, MuRF1, MuRF2 and MuRF3, in 594 unrelated HCM patients and 307 healthy controls by targeted resequencing. Identified rare variants were confirmed by capillary Sanger sequencing. The prevalence of rare variants in both MuRF1 and MuRF2 in HCM patients was higher than that in control subjects (MuRF1 13/594 (2.2%) *vs.* 1/307 (0.3%), *p* = 0.04; MuRF2 22/594 (3.7%) *vs.* 2/307 (0.7%); *p* = 0.007). Patients with rare variants in MuRF1 or MuRF2 were younger (*p* = 0.04) and had greater maximum left ventricular wall thickness (*p* = 0.006) than those without such variants. Mutations in genes encoding sarcomere proteins were present in 19 (55.9%) of the 34 HCM patients with rare variants in MuRF1 and MuRF2. These data strongly supported that rare variants in *MuRF1* and *MuRF2* are associated with higher penetrance and more severe clinical manifestations of HCM. The findings suggest that dysregulation of the ubiquitin-proteasome system contributes to the pathogenesis of HCM.

## 1. Introduction

Hypertrophic cardiomyopathy (HCM) is defined by the presence of left ventricular hypertrophy in the absence of loading conditions sufficient to cause the observed abnormality [1]. It is the most common monogenic cardiovascular disease, caused mainly by mutations in genes encoding sarcomere proteins [2,3,4,5]. Mutations in other genes, such as those encoding *Z*-disc and calcium-handling proteins, are also reported to be associated with the disease, but with low prevalence and less certainty [6].

HCM is characterized by marked clinical heterogeneity [1,7]. There is variable penetrance and variable age of onset, ranging from infancy to old age. The severity of cardiac hypertrophy and left ventricular outflow obstruction vary considerably, and clinical outcomes are heterogeneous. This phenotypic diversity is present even among patients with same genetic mutation [8], suggesting a role for modifier genetic or environmental factors.

A delicate balance between protein synthesis and degradation is necessary for cardiac homeostasis. The ubiquitin-proteasome system (UPS) is responsible for the degradation of 70%–90% intracellular proteins. Impairment of the UPS has been implicated in various cardiac diseases, including HCM [9,10,11,12,13]. Muscle ring finger (MuRF) proteins MuRF1, 2 and 3 (encoded by TRIM63, TRIM55 and TRIM54, respectively) comprise a subfamily of the RING-finger E3 ubiquitin ligases that are specifically expressed in striated muscles [14,15]. They can create complexes as homodimers and heterodimers, and regulate myocyte size and contractility through proteasome-dependent degradation of sarcomere proteins and transduction factors of hypertrophic signaling [16,17,18,19,20,21,22,23]. Recently, mutations in the gene encoding MuRF1 were reported to cause HCM by impairing protein degradation in cardiomyocytes [24], suggesting genetic variants in genes encoding MuRF proteins might be involved in the pathogenesis of HCM. In the present study, we evaluated the association between genetic variants of *MuRF* genes and HCM phenotype by screening all the three members of MuRF subfamily in a large HCM cohort and matched healthy controls.

## 2. Results and Discussion

### 2.1. Results

#### 2.1.1. Rare Variants in *MuRF1* and *MuRF2* Genes

In the *MuRF1* gene, 14 rare non-synonymous variants were identified, including 1 nonsense and 13 missense variants (Table 1). All rare variants were heterozygous and were individual. The rare variants were present in 13 (2.2%) of the 594 HCM patients and 1 (0.3%) of the 307 healthy controls, respectively (Table 2). Rare variants were therefore more prevalent in HCM patients (χ^2^ = 4.6, *p* = 0.04). A known common single nucleotide polymorphism (SNP), K237E, was also detected (Table S1), at comparable frequencies in HCM patients and healthy controls (MAF: 0.23 and 0.19, respectively; χ^2^ = 3.0, *p* = 0.09).

In the *MuRF2* gene, 19 rare nonsynonymous variants were found, including 18 missense, 1 insertion and 1 deletion variant (Table 1). Except for K343R, P392T and T506S, all other rare variants of MuRF2 were unique to single individual. Of the 19 variants, 17 were identified in 22 (3.7%) of the HCM patients and 2 in 2 (0.7%) of the healthy control subjects (Table 2). Therefore, the prevalence of rare variants in HCM patients was significantly higher than that in controls (χ^2^ = 7.3, *p* = 0.007). Two common variants, Q157K and A489V, were detected in this study population (Table S1). These two common variants have comparable frequencies in HCM patients and healthy controls (MAF of Q157K: 0.005 and 0.008, respectively, χ^2^ = 0.64, *p* = 0.52; MAF of A489V: 0.007 and 0.008, respectively, χ^2^ = 0.11, *p* = 0.77).

Taken together, 33 rare variants in the *MuRF1* and *MuRF2* genes were identified in 34 (5.7%) of the HCM patients and 3 (1.0%) of the control subjects (χ^2^ = 11.6, *p* < 0.001; Table 2), including 1 HCM patient who carried rare variants in both genes. Among them, 1 nonsense (*E299X* in *MuRF1*), 3 frameshift (*Q187fs*, *E371fs* and *F538fs* in *MuRF2*) and 14 missense variants were considered pathogenic, based on the expectation of a truncated protein product or a damaging effect predicted by PolyPhen2 (http://genetics.bwh.harvard.edu/pph2/) or SIFT (http://sift.jcvi.org/) (Table 1). Likewise, pathogenic variants in *MuRF1* and *MuRF2* were identified at a greater prevalence in HCM patients than in control subjects (22/594, 3.7% *vs.* 3/307, 1.0%; χ^2^ = 5.6, *p* = 0.02; Table 2).

#### 2.1.2. Rare Variants in *MuRF3* Gene

In the MuRF3 gene, 19 rare variants were identified in 14 (2.4%) of the HCM patients and 8 (2.6%) of the healthy controls (Table 2). All detected variants were missense and heterozygous (Table S2). The frequency of rare variants in *MuRF3* was equal between HCM patients and healthy controls (χ^2^ = 0.053, *p* = 0.82). No common variants in the *MuRF3* gene were detected in the study population.

#### 2.1.3. Coexistence of Sarcomere Mutations with *MuRF1* and *MuRF2* Rare Variants

In order to determine whether the rare variants in genes encoding MuRF1 and MuRF2 were independent causes of HCM, the coexistence of mutations in 8 sarcomere genes was analyzed (Table 1). Among 34 HCM patients with rare variants in the *MuRF1* or the *MuRF2* genes, 19 (55.9%) carried mutations in sarcomere genes. Similarly, among the 22 HCM patients with potentially pathogenic variants in the *MuRF1* or the *MuRF2* genes, sarcomere mutations were present in 12 (54.5%).

#### 2.1.4. Association of *MuRF1* and *MuRF2* Rare Variants with Phenotypes of Hypertrophic Cardiomyopathy (HCM) Patients

The phenotypes of HCM patients were ascertained to determine whether rare variants of *MuRF1* and *MuRF2* modify the clinical expression of the disease (Table 3 and Table S3). We found that patients with rare variants of *MuRF1* and *MuRF2* were younger (44.5 ± 14.2 *vs.* 49.6 ± 14.0 years, *p* = 0.04), and had greater maximum left ventricular wall thickness (23.8 ± 5.2 *vs.* 21.5 ± 4.7 mm, *p* = 0.006) than those without such variants. Likewise, patients with potentially pathogenic rare variants were also younger (*p* = 0.02), with thicker left ventricular wall (*p* = 0.02).

### 2.2. Discussion

Although polymorphisms in genes encoding members of the renin–angiotensin–aldosterone system and other genes implicated in cardiac growth have been reported to be associated with the penetrance and severity of cardiac hypertrophy in HCM [25,26,27], modifier gene effects in HCM are still largely unknown. In the present study, we sequenced genes encoding all three members of *MuRF* E3-liagse subfamily, including *MuRF1*, *MuRF2* and *MuRF3*, in 594 HCM patients and 307 healthy controls, and found that rare nonsynonymous variants in genes encoding *MuRF1* and *MuRF2*, but not *MuRF3*, were more prevalent in HCM patients (respectively, approximately seven times and five times). Patients with these variants were younger and had a greater left ventricular wall thickness. Thus, our findings indicate that rare variants in MuRF1 and MuRF2 can modify the phenotypic expression of HCM.

In most mammalian cells, the UPS controls many fundamental biological processes through targeting the degradation of most cytosolic, nuclear and myofibrillar proteins in a highly regulated manner [9]. Impairment of this process has been observed in various cardiac diseases. In human HCM, proteasome activity is decreased, accompanied by increased mediators of cardiac hypertrophy and apoptosis [28]. Expression of mutant MYPBC3 proteins, one of the most common causes of HCM, results in UPS impairment in both cultured neonatal cardiomyocytes and animal models [11,12,13,29,30]. Furthermore, inhibition of proteasome activity produces cardiac hypertrophy *in vivo*. Thus, the UPS may play an important role in HCM pathogenesis.

The rate-limiting enzyme in UPS-dependent protein degradation is the ubiquitin E3-ligase, which recognizes specific substrates and catalyzes the transfer of activated ubiquitin to them. *MuRF1*, *MuRF2* and *MuRF3* belong to the same E3-ligase subfamily, and are restrictedly expressed in cardiac and skeletal muscles [14,15]. *MuRF1* and *MuRF2* interact with a number of the same sarcomere and sarcomere-related proteins, and are implicated in contractile regulation, myogenic responses and regulation of cardiac hypertrophy [31,32]. Whereas increased expression of *MuRF1* in cardiomyocytes inhibits the development of cardiac hypertrophy [16], deletion of *MuRF1* leads to exaggerated cardiac hypertrophy in response to pressure overload [19]. Although mice with deletion of either *MuRF-1* or *MuRF-2* alone exhibit a normal cardiac phenotype at baseline, *MuRF-1*/*MuRF-2* double knockout mice develop massive spontaneous hypertrophic cardiomyopathy [31].

Impairment of the UPS may underlie the association of rare variants in the genes encoding *MuRF1* and *MuRF2* with increased risk for HCM and greater severity of phenotypic expressions observed in our study. Variants in these two genes may reduce protein degradation through the UPS, and thereby lead to accumulation of mutant proteins and cardiac hypertrophy regulators. Consistent with our findings, previous studies have shown that expression of mutant MYBPC3 protein in an HCM model is markedly increased by proteasome inhibition [30]. In a recent study, cardiac-specific over-expression of *MuRF1* mutations led to increased sarcomere protein expressions and activation of the MTOR-S6K and calcineurin pathways [24].

**Table 1:** Rare nonsynonymous variants identified in *MuRF1* and *MuRF2* genes.

Gene	cDNA	Protein	Type	PP2 *	SIFT ^†^	Pathogenic ^§^	Patients	Controls	Sarcomere Mutations ^‡^
*MuRF1*	*c.14C>T*	S5L	ms	pro (0.985)	T (0.10)	pathogenic	1	0	n/d
*NM*_*032588*	*c.183C>A*	S61R	ms	ben (0.079)	T (0.17)	benign	1	0	*MYH7*, *K1757E*
	*c.218T>C*	F73S	ms	pro (1)	T (0.23)	pathogenic	1	0	n/d
	*c.256C>T*	R86C	ms	pro (0.98)	D (0.01)	pathogenic	1	0	n/d
	*c.257G>A*	R86H	ms	pro (1)	D (0.05)	pathogenic	1	0	*MYBPC3*, *E258K*
	*c.301A>T*	I101F	ms	pro (0.987)	D (0.01)	pathogenic	1	0	n/d
	*c.378A>T*	E126D	ms	ben (0.216)	T (0.24)	benign	1	0	n/d
	*c.695C>T*	T232M	ms	pro (0.99)	T (0.05)	pathogenic	1	0	*MYBPC3*, *c.3491-1G>A*
	*c.760G>A*	D254N	ms	ben (0.161)	T (0.21)	benign	1	0	*MYH7*, *K1242fs*
	*c.895G>T*	E299 *	ns	–	–	pathogenic	1	0	*MYL3*, *E49D*
	*c.915G>T*	M305I	ms	pos (0.898)	T (0.38)	pathogenic	0	1	–
	*c.953C>A*	A318D	ms	pos (0.602)	D (0.03)	pathogenic	1	0	*MYH7*, *R453C*
	*c.962C>A*	A321D	ms	ben (0)	T (0.53)	benign	1	0	*MYBPC3*, *R160W*
	*c.1051G>A*	G351W	ms	ben (0.001)	T (0.25)	benign	1	0	*MYL2*, *R58Q*
*MuRF2*	*c.149G>A*	C50Y	ms	pro (1)	D (0)	pathogenic	1	0	n/d
*NM*_*033058*	*c.160A>G*	I54V	ms	ben (0.088)	T (0.24)	benign	1	0	*MYH7*, *V1360I*
	*c.235C>G*	P79A	ms	ben (0.446)	D (0)	pathogenic	1	0	*MYH7*, *Q892K*
	*c.558_559insA*	Q187fs	fs	–	–	pathogenic	1	0	*MYH7*, *K1757E*
	*c.721C>A*	L241M	ms	pro (0.994)	D (0)	pathogenic	1	0	n/d
	*c.755C>T*	S252F	ms	pos (0.726)	D (0)	pathogenic	0	1	–
	*c.771C>A*	N257K	ms	ben (0.015)	T (0.73)	benign	1	0	*MYH7*, *G741R*
	*c.772G>A*	V258I	ms	ben (0.32)	T (0.34)	benign	1	0	n/d
	*c.1006G>C*	E336Q	ms	ben (0.09)	T (0.22)	benign	1	0	n/d
	*c.1028A>G*	K343W	ms	ben (0)	T (0.47)	benign	2	0	*MYBPC3*, *Y842X*; n/d
	*c.1112delA*	E371fs	fs	–	–	pathogenic	1	0	*MYBPC3*, *c.1928-2A>G*
	*c.1174C>A*	P392T	ms	pos (0.546)	D (0.01)	pathogenic	4	0	*MYH7*, *R663H*; *MYBPC3*, *K301fs*; *MYH7*, *R652S*; n/d
	*c.1253C>T*	T418I	ms	ben (0.001)	T (0.13)	benign	1	0	n/d
	*c.1356A>T*	K452N	ms	pos (0.651)	D (0.03)	pathogenic	1	0	n/d
	*c.1373C>T*	P458L	ms	ben (0)	T (0.25)	benign	1	0	n/d
	*c.1462G>A*	A488T	ms	poss (0.553)	T (0.19)	pathogenic	0	1	–
	*c.1516A>T*	T506S	ms	pos (0.666)	T (0.41)	pathogenic	2	0	*MYH7*, *R204H*; n/d
	*c.1568A>G*	H523W	ms	ben (0.003)	D (0)	pathogenic	1	0	*MYBPC3*, *R1037C*
	*c.1614delC*	F538fs	fs	–	–	pathogenic	1	0	n/d

Abbreviations: fs, frame-shift variant; ms, missense variant; ns, nonsense variant; n/d, no sarcomere mutation detected; * Pathogenicity and scores of missense variants predicted by PolyPhen2; Pro, probably damaging; pos, possible damaging; ben, benign; **^†^** Pathogenicity and scores of missense variants predicted by SIFT; D, deleterious; T, tolerated; **^§^** Nonsense and frame-shift variants were considered to be pathogenic as they were expected to result in truncated proteins; The pathogenic missense variant is defined by a damaging effect predicted by either PolyPhen2 (http://genetics.bwh.harvard.edu/pph2/) or SIFT (http://sift.jcvi.org/); **^‡^** All the sarcomere mutations listed are pathogenic.

**Table 2:** Prevalence of *MuRF* rare variants in patients with hypertrophic cardiomyopathy and healthy control subjects.

Gene	Rare Variants				Pathogenic Rare Variants			
	Patients (*n* = 594)	Controls (*n* = 307)	χ^2^	*p* Value	Patients (*n* = 594)	Controls (*n* = 307)	χ^2^	*p* Value
*MuRF1*	13 (2.2%)	1 (0.3%)	4.6	0.04	8 (1.3%)	1 (0.3%)	2.1	0.18
*MuRF2*	22 (3.7%)	2 (0.7%)	7.3	0.007	14 (2.4%)	2 (0.7%)	3.4	0.11
*MuRF3*	14 (2.4%)	8 (2.6%)	0.053	0.82	6 (1.00%)	4 (1.3%)	0.16	0.74
*MuRF1* and *MuRF2*	34 (5.7%)	3 (1.0%)	11.6	<0.001	22 (3.7%)	3 (1.0%)	5.6	0.02

**Table 3:** Correlation of pathogenic rare variants in *MuRF1* and *MuRF2* genes to the clinical manifestations of patients with hypertrophic cardiomyopathy.

Clinical Manifestations	Total (*n* = 594)	Pathogenic Rare Variant		
		With (*n* = 22)	Without (*n* = 572)	*p* Value
Age (year)	49.3 ± 14.1	42.6 ± 13.2	49.6 ± 14.0	0.02
Female (No.)	183 (30.8%)	7 (31.8%)	176 (30.8%)	1.0
Height (cm)	167.1 ± 8.0	168.4 ± 8.8	167.1 ± 8.0	0.46
Weight (kg)	71.1 ± 11.9	69.3 ± 9.4	71.1 ± 12.0	0.49
FH of HCM (No.)	133 (22.4%)	5 (22.7%)	128 (22.4%)	1.0
FH of SCD (No.)	76 (12.8%)	1 (4.5%)	75 (13.1%)	0.34
Heart rate (bpm)	71.0 ± 12.0	70.8 ± 15.6	71.0 ± 11.8	0.93
Abnormal Q wave (No.)	133 (22.4%)	6 (27.3%)	127 (22.2%)	0.60
Abnormal T wave (No.)	396 (66.7%)	17 (77.3%)	379 (66.3%)	0.36
NYHA class III or IV (No.)	68 (11.4%)	2 (9.1%)	66 (11.5%)	1.0
Maximum LV wall thickness (mm)	21.6 ± 4.7	24.0 ± 5.6	21.6 ± 4.7	0.02
LV end diastolic diameter (mm)	44.9 ± 6.1	42.3 ± 6.2	45.0 ± 6.0	0.09
LV ejection fraction (%)	66.6 ± 8.8	64.7 ± 8.9	66.7 ± 8.8	0.31
LV outflow obstruction (No.) *	230 (38.7%)	9 (40.9%)	221 (38.6%)	0.83
Left atrium size (mm)	40.1 ± 6.8	39.0 ± 8.2	40.1 ± 6.8	0.44

Abbreviations: FH, family history; HCM, hypertrophic; cardiomyopathy; LV, left ventricular; NYHA, New York Heart; Association; SCD, sudden cardiac death; * Defined as left ventricular outflow tract gradient ≥30 mmHg at resting.

*MuRF3* appears to have a different function from *MuRF1* and *MuRF2*. *MuRF3* mainly interacts with microtubule proteins, whereas *MuRF1* and *MuRF2* interact with sarcomeric contractile proteins [18]. This functional difference may explain why variants in the *MuRF3* gene were not associated with HCM in our study.

Recently, Chen *et al.* reported mutations in the gene encoding *MuRF1* as a likely cause of HCM [24]. They identified three mutations in the *MuRF1* gene in 5 of 302 HCM probands. These mutations were absent in 1090 controls and coexistence of mutations in HCM-causing sarcomere genes were excluded. Although the size of families in was not sufficient for genetic linkage analysis, transgenic over-expression of any of the three mutations led to cardiac hypertrophy in mice, suggesting *MuRF1* mutations are independent causes of HCM. Nevertheless, controversy remains since other study found that individuals with MuRF1 p.Q247X, one of the disease-causing mutations, reported by Chen *et al.* [24], had no evidence of HCM [33]. In our study, rare variants in the *MuRF1* and *MuRF2* genes were present in healthy control subjects, albeit at a much lower prevalence than in HCM patients. Bioinformatics analysis predicted that the rare variants identified in controls were damaging. More importantly, sarcomere mutations were identified in more than half of the HCM patients with rare variants in the *MuRF1* and *MuRF2* genes, comparable to its prevalence in the general HCM population. Such a high frequency of coexisting sarcomere mutations and *MuRF* variants suggests that most rare MuRF variants were modifiers and not independent causes of HCM.

Limitation: First, the frequency of MuRF rare variants in healthy sarcomere-mutation carriers was not analyzed in the present study. A comparison of the prevalence of *MuRF* rare variants between HCM patients with sarcomere mutations and healthy sarcomere-mutation carriers would help to confirm the modifier effect of rare variants in *MuRF1* and *MuRF2* genes on the development and phenotypic expression of HCM; Second, in the present study, although more than 50% of the patients either with rare variants or with pathogenic rare variants of *MuRF1* and *MuRF2* genes carried mutations of sarcomere genes, the HCM-causing roles of these variants, especially those identified in patients without sarcomere mutation detected, cannot be excluded completely. Finally, the number of patients with rare variants of *MuRF1* and *MuRF2* is relatively small. The findings in our study require validation in other large cohorts, and should be interpreted with caution.

## 3. Experimental Section

### 3.1. Ethics Statement

This study was performed in accordance with the principle of the Declaration of Helsinki and approved by the Ethics Committees of Fuwai Hospital. All participants provided written informed consent.

### 3.2. Study Subjects

A total of 594 unrelated HCM patients and 307 age- and sex-matched healthy controls were included in this study. HCM was defined by an unexplained maximal left ventricular wall thickness ≥15 mm on echocardiography or cardiac magnetic resonance imaging in the absence of another cardiac or systemic disease capable of producing that magnitude of hypertrophy. None of the control subjects had a history of cardiovascular or other systemic diseases. Normal cardiac structure and function in healthy controls was confirmed by evaluation with a 12-lead electrocardiogram and an echocardiogram.

### 3.3. Gene Sequencing and Variant Classification

Genomic DNA from all HCM and control subjects was isolated from peripheral blood leukocytes and used to construct shotgun libraries of approximately 250 bp fragments with index adaptors. All coding exons of the genes encoding *MuRF1*, *MuRF2* and *MuRF3* and their adjacent 5 bp intronic sequences were enriched by using a custom designed probe library (Agilent Technologies, Santa Clara, CA, USA), and sequenced on Illumina GAIIx (Illumina Inc., San Diego, CA, USA) to generate pair-end sequencing reads of 120 bp at each end. After removal of PCR duplications with PICARD (http://picard.sourceforge.net/), sequencing reads of each individual were mapped to the human genome (*GRCh37/hg19*) with CLC Genomics Workbench (CLC-bio, Aarhus, Denmark). The sequencing depth was analyzed and variants in *MuRF* genes were called using the following filter parameters: coverage ≥25× and variant frequency ≥20%. Synonymous variants were excluded from further analysis. A rare variant was defined as one present with a minor allele frequency (MAF) of <0.5% in the studied subjects. All rare nonsynonymous variants identified by targeted resequencing were confirmed by bidirectional capillary Sanger sequencing. Eight sarcomere genes (*MYH7*, *MYBPC3*, *TNNT2*, *TNNI3*, *MYL2*, *MYL3*, *TPM1* and *ACTC1*) were also evaluated by targeted resequencing in HCM subjects.

A rare missense variant was considered to be potentially pathogenic when predicted to be damaging by any of PolyPhen-2 [34] or SIFT programs [35]. Nonsense and frame-shift variants were considered to be pathogenic since they were expected to result in truncated proteins.

### 3.4. Statistical Analysis

Continuous variables are presented as mean ± standard deviation and categorical variables as frequencies (n) and percentages (%). The Student’s *t* test was used for the comparison of continuous variables, and χ^2^ or Fisher exact test for non-continuous variables. *p* values are two-sided and considered significant when <0.05. Calculations were performed using PASW Statistics 18 software (SPSS, Chicago, IL, USA).

## 4. Conclusions

In conclusion, rare variants in the genes encoding *MuRF1* and *MuRF2* increase the risk of development of HCM and lead to more severe disease phenotypes, and may act as modifier of the disease. Our study is consistent with the hypothesis that impaired ubiquitin-proteasome system contributes to the pathogenesis of HCM.

## Supplementary Files

Supplementary File 1 Supplementary Information (PDF, 155 KB)
