# Supplementary material for: Rare Variants in Genes Encoding MuRF1 and MuRF2 Are Modifiers of Hypertrophic Cardiomyopathy
Source: Int J Mol Sci. 2014 May 26;15(6):9302–13. doi: 10.3390/ijms15069302 (PMC4100095; doi:10.3390/ijms15069302)
Supplement: Supplementary File 1 — Supplementary Information (PDF, 155 KB) [file ijms-15-09302-s001.pdf]

Supplementary Information

Table S1. Common nonsynonymous variants detected in the studied population.

Gene	Variant	Genotype	HCM Patients (n = 594)	Healthy Controls (n = 307)	χ^2	p Value
MuRF1	*K237E*	KK	354 (59.6)	200 (65.1)		
NM_032588		KE	212 (35.7)	97 (31.6)		
		EE	28 (4.7)	10 (3.3)		
		MAF	0.23	0.19	3.0	0.09
MuRF2	*Q157K*	QQ	588 (99.0)	302 (98.4)		
NM_033058		QK	6 (1.0)	5 (1.6)		
		MAF	0.007	0.008	0.64	0.52
	A489V	AA	586 (98.7)	302 (98.4)		
		AV	8 (1.3)	5 (1.6)		
		MAF	0.007	0.008	0.11	0.77

Data were presented as number (percentage).

Table S2. Rare nonsynonymous variants identified in the *MuRF3* gene.

Gene	cDNA	Protein	Patients	Controls	PP2 *	SIFT †	Pathogenic §
MuRF3	*c.26C>T*	P9L	0	1	pro (0.997)	T (0.12)	pathogenic
NM_*032546*	*c.160G>A*	V54I	1	0	ben (0.025)	T (1.00)	benign
	c.280G>T	G94C	1	0	pro (1)	D (0)	pathogenic
	c.343C>T	P115S	0	1	pos (0.521)	T (0.08)	pathogenic
	c.488T>C	L163P	0	1	pro (1)	T (0.17)	pathogenic
	c.587C>T	P196L	1	1	ben (0)	T (0.31)	benign
	c.607A>G	I203V	1	0	ben (0.001)	T (0.29)	benign
	c.662C>T	A221V	1	0	pro (0.999)	T (0.27)	pathogenic
	c.746G>A	R249Q	0	1	pos (0.582)	T (0.44)	pathogenic
	c.806G>A	R269H	1	0	pro (1)	D (0.01)	pathogenic
	c.810G>T	K270N	1	0	pro (0.999)	D (0)	pathogenic
	c.859C>A	R287S	1	0	ben (0.033)	T (0.86)	benign
	c.860G>T	R287L	1	0	ben (0.005)	T (0.69)	benign
	c.1015G>A	E339K	2	1	ben (0.051)	T (0.29)	benign
	c.1036C>A	P346T	1	0	pos (0.758)	T (0.19)	pathogenic
	c.1066G>T	V356L	0	1	ben (0.023)	T (0.88)	benign
	c.1087G>A	E363K	0	1	ben (0.002)	T (0.87)	benign
	c.1118G>A	G373D	1	0	pro (0.999)	T (0.39)	pathogenic
	c.1147C>A	P383T	1	0	ben (0.066)	T (0.32)	benign

* Pathogenicity and scores of missense variants predicted by PolyPhen2; Pro, probably damaging; pos, possible damaging, and ben means benign; † Pathogenicity and scores of missense variants predicted by SIFT; D, deleterious; T, tolerated; § The pathogenic missense variant is defined by a damaging effect predicted by either PolyPhen2 or SIFT.

Table S3. Correlation of rare variants in *MuRF1* and *MuRF2* genes to the clinical manifestations of patients with hypertrophic cardiomyopathy.

Clinical Manifestations	Total (n = 594)	Rare Variant		
		With (n = 34)	Without (n = 560)	p Value
Age (year)	49.3 ± 14.1	44.5 ± 14.2	49.6 ± 14.0	0.04
Female (No.)	183 (30.8%)	13 (38.2%)	170 (30.4%)	0.34
Height (cm)	167.1 ± 8.0	166.8 ± 8.9	167.1 ± 8.0	0.81
Weight (kg)	71.1 ± 11.9	68.7 ± 10.1	71.2 ± 12.0	0.23
FH of HCM (No.)	133 (22.4%)	11 (32.4%)	122 (21.8%)	0.20
FH of SCD (No.)	76 (12.8%)	5 (14.7%)	71 (12.7%)	0.79
Heart rate (bpm)	71.0 ± 12.0	69.4 ± 12.5	71.1 ± 11.9	0.43
Abnormal Q wave (No.)	133 (22.4%)	10 (29.4%)	123 (22.0%)	0.30
Abnormal T wave (No.)	396 (66.7%)	23 (67.6%)	373 (66.6%)	1.0
NYHA class III or IV (No.)	68 (11.4%)	5 (14.7%)	63 (11.2%)	0.58
Maximum LV wall thickness (mm)	21.6 ± 4.7	23.8 ± 5.2	21.5 ± 4.7	0.006
LV end diastolic diameter (mm)	44.9 ± 6.1	43.5 ± 6.2	45.0 ± 6.0	0.16
LV ejection fraction (%)	66.6 ± 8.8	64.5 ± 10.3	66.7 ± 8.7	0.15
LV outflow obstruction (No.) *	230 (38.7%)	11 (32.4%)	219 (39.1%)	0.47
Left atrium size (mm)	40.1 ± 6.8	39.6 ± 7.2	40.1 ± 6.8	0.68

Abbreviations: FH, family history; HCM, hypertrophic cardiomyopathy; LV, left ventricular; NYHA, New York Heart Association; SCD, sudden cardiac death; * Defined as left ventricular outflow tract gradient ≥30 mmHg at resting.

© 2014 by the authors; licensee MDPI, Basel, Switzerland. This article is an open access article distributed under the terms and conditions of the Creative Commons Attribution license (http://creativecommons.org/licenses/by/3.0/).
